# Rosuvastatin in Combination With Bortezomib Promotes Osteogenesis in Myeloma Bone Disease by Inhibiting the Secretion of CCL3

**DOI:** 10.1002/jha2.70232

**Published:** 2026-01-28

**Authors:** Fujun Qu, Mingxu Hui, Fan Yang, Bo Huang, Nian Liu, Yuchan He, Xiaotao Wang

**Affiliations:** ^1^ Department of Hematology The First Affiliated Hospital of Guilin Medical University Guilin Guangxi China; ^2^ Department of Radiation Oncology The Second Affiliated Hospital of Guilin Medical University Guilin Guangxi China; ^3^ Department of Pathology The First Affiliated Hospital of Guilin Medical University Guilin Guangxi China; ^4^ Department of Hematology The Second Affiliated Hospital of Guilin Medical University Guilin Guangxi China; ^5^ Health Management Center The Second Affiliated Hospital of Guilin Medical University Guilin Guangxi China

**Keywords:** Bortezomib, CCL3, MBD, MM, Rosuvastatin

## Abstract

**Background:**

Myeloma bone disease (MBD) is a devastating complication of multiple myeloma (MM) and a primary cause of disability in affected patients. Its pathogenesis is driven by an imbalance in bone remodeling, largely attributed to the overexpression of C‐C motif chemokine ligand 3 (CCL3). While both bortezomib and statins have been reported to inhibit CCL3, their combined effect on promoting osteogenesis in MBD remains unexplored. This study aimed to investigate the therapeutic potential and underlying mechanism of rosuvastatin in combination with bortezomib for MBD.

**Methods:**

Bone marrow samples from MBD patients were analyzed to correlate CCL3 levels with bone turnover markers. Human myeloma cell lines (IM9, XG‐1) were treated with bortezomib and/or rosuvastatin. Cell viability was assessed by CCK‐8 assay, and CCL3 expression was measured by ELISA and Western blot. A NOD/SCID mouse model of MBD was established to evaluate the in vivo effects on tumor growth, CCL3 secretion (by immunohistochemistry), and bone remodeling (by ALP and TRAP double staining).

**Results:**

CCL3 levels were significantly elevated in MBD patients and positively correlated with the severity of bone destruction. The combination of bortezomib and rosuvastatin synergistically inhibited CCL3 secretion in vitro and in vivo more effectively than either monotherapy. Consequently, the combination treatment significantly enhanced osteoblast activity, suppressed osteoclast formation, and improved survival in the murine model.

**Conclusion:**

The combination of rosuvastatin and bortezomib exerts a synergistic effect by inhibiting CCL3, thereby rebalancing bone remodeling and promoting osteogenesis. This strategy represents a promising novel therapeutic approach for mitigating MBD.

## 1 Introduction

Multiple myeloma (MM) ranks as the second most prevalent hematologic malignancy [1]. A hallmark of MM is myeloma bone disease (MBD), which affects up to 80% of patients at the time of diagnosis [2]. MBD is frequently linked with severe pain, pathological fractures, spinal cord compression, vertebral collapse, and hypercalcemia [3], all of which constitute the primary contributors to disability in MM patients. Despite advancements in treatment, including the use of lenalidomide, bortezomib, thalidomide, and autologous stem cell transplantation, which have markedly enhanced progression‐free survival (PFS) and overall survival (OS), approximately 40% of individuals with MBD ultimately succumb to the disease. Consequently, the paramount objective is the effective control and management of bone disease to improve the prognosis for patients with MBD.

The pathogenesis of MBD is characterized by an imbalance in the bone remodeling process, marked by the activation of osteoclasts (OCs), suppression of osteoblasts (OBs), and an immunosuppressive bone marrow microenvironment [4]. Within this microenvironment, myeloma cells (MCs) and bone marrow stromal cells (BMSCs), alongside OBs and OCs, secrete various cytokines that facilitate bone resorption and reconstruction [5]. C‐C motif chemokine ligand 3 (CCL3), produced by MCs, not only mediates survival and proliferation of MM cells but also plays a pivotal role in the development of osteolytic bone lesions associated with MM [6, 7, 8, 9]. In 2016, Minarik et al. demonstrated that CCL3 is associated with MM activity and the presence of lytic bone lesions [10]. Both bortezomib and statins have been shown to inhibit CCL3 levels, as corroborated by international researchers [11, 12, 13]. However, it remains uncertain whether bortezomib and statins can influence the expression of OBs and OCs through the inhibition of CCL3 expression. Therefore, this study was designed to specifically investigate the effects of bortezomib and rosuvastatin on bone remodeling in MBD, focusing on their role in regulating CCL3 to elucidate a potential synergistic mechanism of action.

## 2 Materials and Methods

### 2.1 Patients and Tissues

Bone marrow samples were collected from 53 patients with MBD and 30 patients with immune thrombocytopenia (ITP) at The Second Affiliated Hospital of Guilin Medical University. Written informed consent was obtained from all participants. This study received approval from the Ethics Committee of The Second Affiliated Hospital of Guilin Medical University. All bone marrow samples were stored at −80°C until further analysis. Inclusion criteria: (1) The study included randomized trials of patients with MBD receiving bortezomib either as monotherapy or in combination with other regimens. (2) The diagnosis of ITP was established in accordance with the Chinese Guidelines for the Diagnosis and Management of Adult Primary ITP. Exclusion criteria: (1) Patients were excluded if they presented with comorbid conditions, including chronic obstructive pulmonary disease, bronchial asthma, or severe infectious diseases. (2) Patients with a prior medical history of malignant tumors were excluded.

### 2.2 Cell Culture and CCK‐8 Assay

Human MM cell lines, IM9 and XG‐1, were obtained from Nanjing Cobioer Biotechnology Co. Ltd. (Nanjing, China). The cells were cultured in RPMI 1640 medium (Hyclone, Logan, UT, USA) supplemented with 10% fetal bovine serum (FBS) (Sigma, St. Louis, MO, USA). The cultures were maintained at 37°C in a humidified atmosphere containing 5% CO_2_. Cell viability was evaluated using the Cell Counting Kit‐8 (CCK‐8) assay. Specifically, IM9 and XG‐1 cells were seeded at a density of 7 × 10^3^ cells per well in 96‐well plates and allowed to adhere for 24 h, followed by treatment with bortezomib or rosuvastatin and incubation for an additional 1–3 days. The specific procedures for optical density (OD) detection have been previously described [14].

### 2.3 ELISA Assay

CCL3 protein levels were quantified using a Human CCL3 ELISA Kit (Cusabio, Wuhan, China). Absorbance measurements were performed at 450 nm using a microplate reader (Tecan, Männedorf, Switzerland). All measurements were performed in triplicate. The analyses were conducted at the Central Laboratory of The Second Affiliated Hospital of Guilin Medical University, Guilin, China.

### 2.4 Western Blot Analysis

Western blotting assays were conducted as previously described [14]. Primary antibodies against CCL3 (Abcam, Cambridge, UK) and β‐actin (ZSGB‐BIO, Beijing, China) were used following the advised dilution.

### 2.5 Construction of the Mouse Xenograft Model

The experiments conducted in this study were approved by the Animal Research Committee of Guilin Medical University. All animal experiments adhered to the National Institutes of Health Guidelines for the Care and Use of Laboratory Animals. The nude mice used in this study were housed in microisolator cages under sterile conditions.

NOD/SCID mice (4–6 weeks old) received intratibial injections of 5 × 10^6^ IM9 cells in the right leg, while control mice received PBS (Solarbio, Beijing, China). Two weeks post‐injection, mice were randomized into four treatment groups: (1) PBS control (7 days); (2) rosuvastatin (10 mg/kg/day for 7 days); (3) bortezomib (2 mg/kg every 2 days); and (4) combination of bortezomib (2 mg/kg every 2 days) and rosuvastatin (10 mg/kg/day for 7 days). Successful establishment of the xenograft model was confirmed using immunohistochemistry (IHC) and mammography.

#### 2.5.1 Immunohistochemistry

Following completion of the xenograft study, mice were euthanized, and tumors were excised. Immunohistochemical staining was carried out according to previously established protocols [14]. Primary antibodies against CCL3 (Affinity, Changzhou, China), CD19 (ZSGB‐BIO, Beijing, China), and CD20 (Maxim Biotechnologies, Fuzhou, China) were used following the advised dilution. Quantitative analysis of IHC was performed using the H‐score system. The formula is: H‐score = 0 × (negative cell percentage) + 1 × (weakly positive cell percentage) + 2 × (moderately positive cell percentage) + 3 × (strongly positive cell percentage), with a score range of 0–300.

### 2.6 Statistical Analysis

Statistical analyses were performed using SPSS 20.0 and GraphPad Prism 10 (GraphPad Software, San Diego, CA, USA). Differences between two groups were analyzed using two‐tailed Student's *t*‐tests. One‐way analysis of variance (ANOVA) was used for multiple group comparisons. All experiments were performed in triplicate. Statistical significance was defined as *p *< 0.05.

## 3 Results

### 3.1 CCL3 is Elevated in MBD Patients and Correlates With Disease Severity and Treatment Response

**FIGURE 1:**
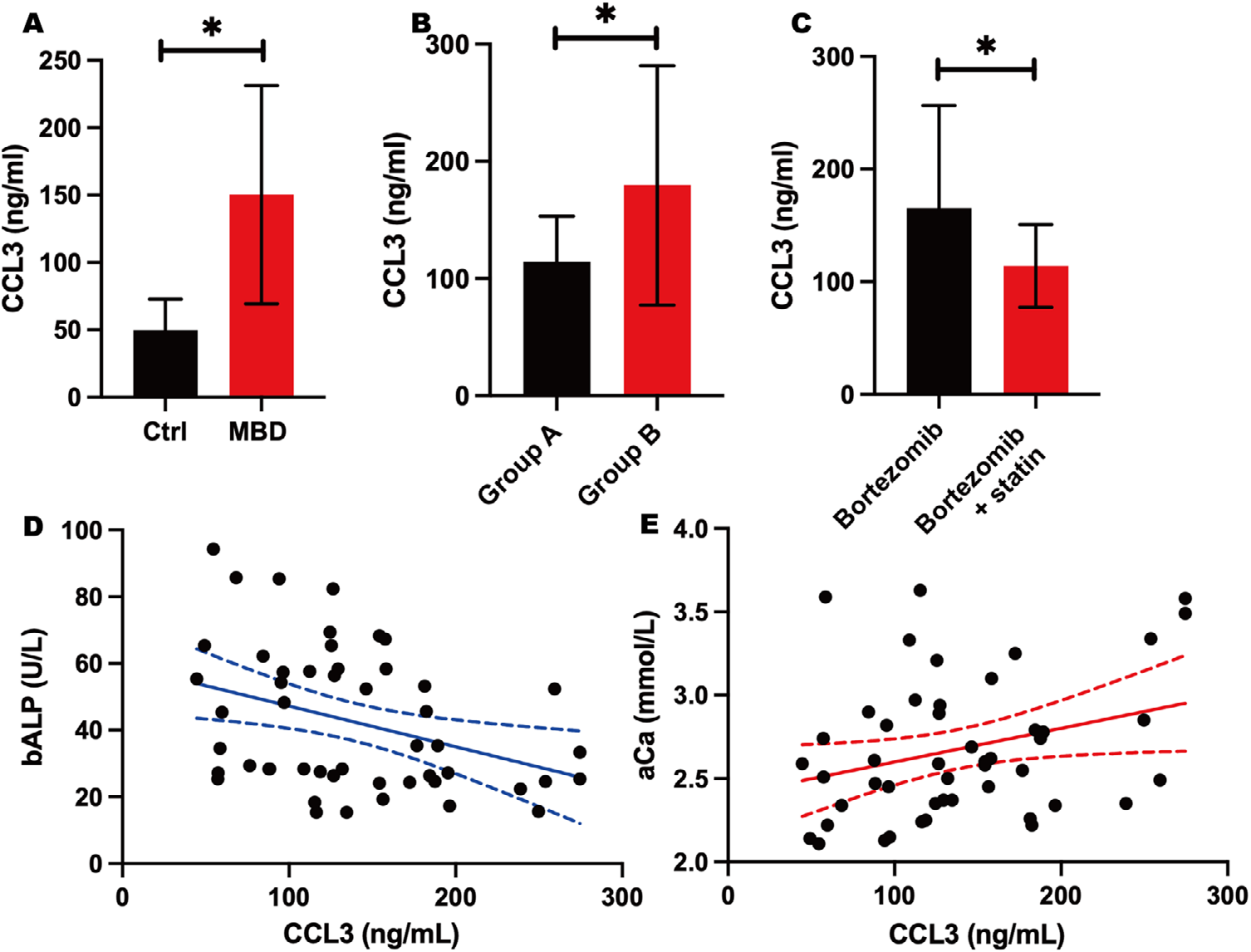
CCL3 is elevated in multiple myeloma bone disease (MBD) patients and correlates with disease severity. (A) Comparison of serum CCL3 levels between MBD patients (150.34 ± 81.08 ng/mL, *n* = 53) and non‐myeloma bone disease controls (49.76 ± 23.01 ng/mL, *n* = 30), as determined by ELISA. Data are presented as mean ± SD. *p < 0.001. (B) Serum CCL3 levels in MBD patients stratified by the number of osteolytic lesions: Group A (< 3 lesions) (114.36 ± 38.73 ng/mL, *n* = 19) and Group B (≥ 3 lesions) (179.52 ± 102.05 ng/mL, *n* = 34). Data are presented as mean ± SD. *p = 0.0074. (C) CCL3 levels in bone marrow plasma from MBD patients treated with Bortezomib alone (165.43 ± 91.19 ng/ml, *n* = 37) versus a combination of Bortezomib and statins (114.06 ± 36.69 ng/mL, *n* = 16). Data are presented as mean ± SD. *p = 0.0348. (D) Negative correlation between serum CCL3 levels and bone‐specific alkaline phosphatase (bALP) in MBD patients (*n* = 53). The solid line represents the linear regression fit (*Y* = −0.122*X* + 59.43), with the 95% confidence interval indicated by the dashed lines. *p* = 0.0111. (E) Positive correlation between serum CCL3 levels and adjusted calcium (aCa) levels in MBD patients (*n* = 53). The solid line represents the linear regression fit (*Y* = 0.002018*X* + 2.0398), with the 95% confidence interval indicated by the dashed lines. *p* = 0.0403.

To elucidate the role of CCL3 in MBD, we first measured serum CCL3 levels in patients. ELISA analysis revealed that CCL3 concentrations in MBD patients (150.34 ± 81.08 ng/mL, *n* = 53) were significantly higher than those in non‐MBD controls (49.76 ± 23.01 ng/mL, *n* = 30) (*p *< 0.001) (Figure 1A), suggesting CCL3 may be involved in the pathological process of MBD.

To investigate the correlation between CCL3 levels and bone destruction severity, we stratified MBD patients based on the number of osteolytic lesions. Patients with ≥ 3 lesions (Group B) exhibited significantly higher serum CCL3 levels (179.52 ± 102.05 ng/mL, *n* = 34) compared to those with < 3 lesions (Group A, 114.36 ± 38.73 ng/mL, *n* = 19) (*p* = 0.0074) (Figure 1B), indicating a positive correlation between CCL3 expression and radiographic bone disease severity.

We further evaluated the impact of drug therapy on CCL3 levels in the bone marrow microenvironment. Analysis of bone marrow plasma showed that patients receiving bortezomib combined with statins (114.06 ± 36.69 ng/mL, *n* = 16) had significantly lower CCL3 levels than those receiving bortezomib monotherapy (165.43 ± 91.19 ng/mL, *n* = 37) (*p* = 0.0348) (Figure 1C), suggesting that combination therapy may more effectively suppress CCL3 production.

To explore the relationship between CCL3 and bone metabolism biomarkers, correlation analyses were performed. Serum CCL3 levels showed a significant negative correlation with bone‐specific alkaline phosphatase (bALP) (*p* = 0.0111) (Figure 1D). Conversely, a significant positive correlation was observed between serum CCL3 and albumin‐corrected calcium (aCa) levels (*p* = 0.0403) (Figure 1E). These clinical data suggest a potential role for CCL3 in disrupting bone remodeling in MBD.

### 3.2 Rosuvastatin Enhances the Anti‐Myeloma Effect of Bortezomib and Suppresses CCL3 Expression In Vitro

**FIGURE 2:**
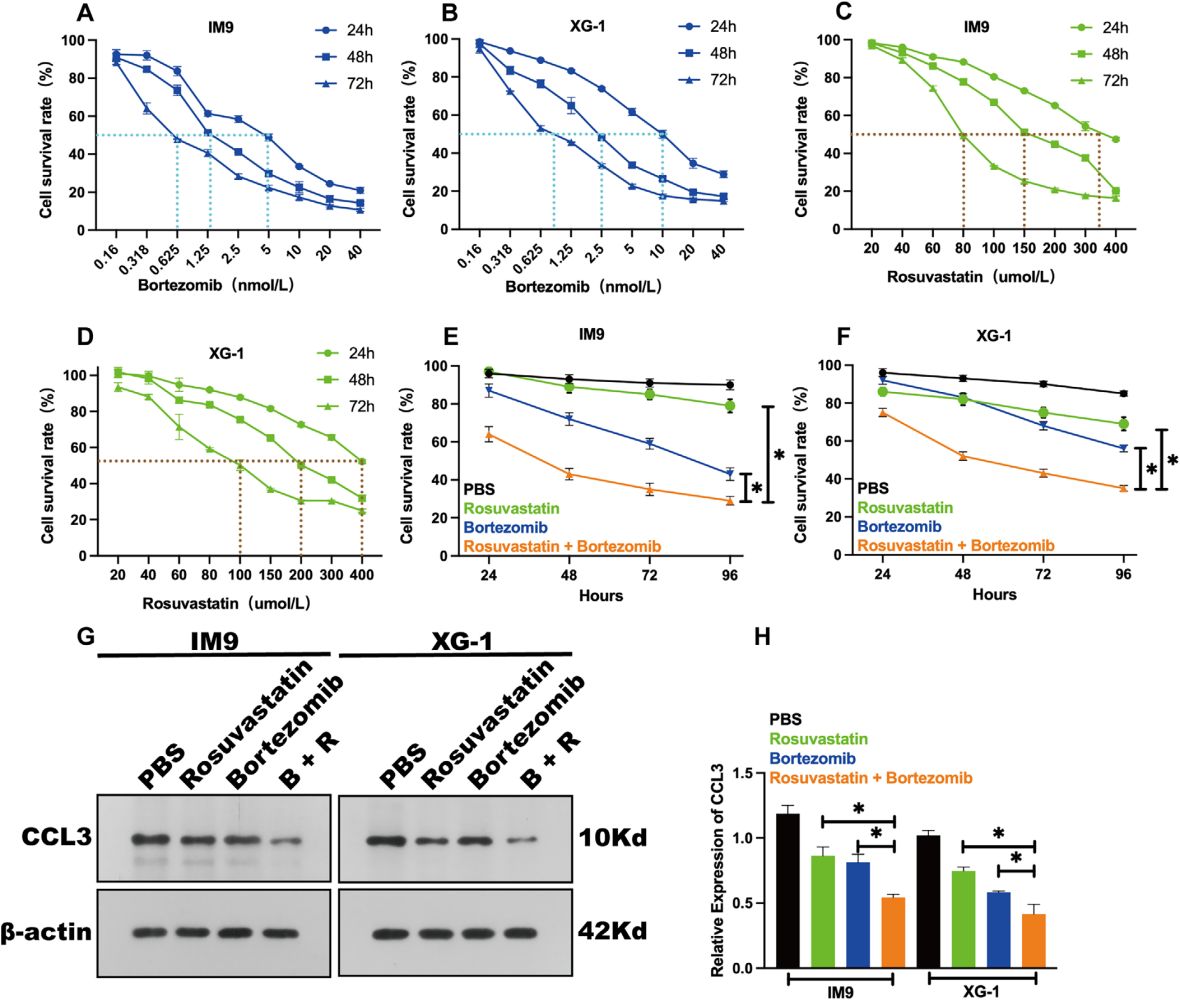
Bortezomib and rosuvastatin synergistically inhibit proliferation and CCL3 expression in myeloma cells. (A–D) Dose‐response curves of bortezomib and rosuvastatin in myeloma cell lines. Viability of IM9 (A, C) and XG‐1 (B, D) cells treated with increasing concentrations of bortezomib (A, B) or rosuvastatin (C, D) for 24, 48, and 72 h was assessed by CCK‐8 assay. Data are presented as the percentage of cell survival relative to the PBS‐treated control group. The dashed lines indicate the half‐maximal inhibitory concentration (IC_50_) for each corresponding time point. (E, F) Combinatorial effects of bortezomib and rosuvastatin on cell proliferation. IM9 (E) and XG‐1 (F) cells were treated with PBS, bortezomib alone (1.25 nM for IM9; 2.5 nM for XG‐1), rosuvastatin alone (150 µM for IM9; 200 µM for XG‐1), or the combination of both drugs for 24, 48, 72, and 96 h. Cell viability was measured by CCK‐8 assay. Data are shown as mean ± SD (*n* = 5 independent experiments). *p < 0.05 compared to the single‐agent groups. (G, H) Bortezomib and rosuvastatin co‐treatment suppresses CCL3 protein expression. (G) Representative Western blot images showing CCL3 protein levels in IM9 and XG‐1 cells after 48‐h treatments with PBS, bortezomib, rosuvastatin, or their combination (using the same concentrations as in E and F). β‐Actin was used as an internal loading control. (H) Densitometric quantification of CCL3 protein expression levels from (G), normalized to β‐actin. Data are presented as mean ± SD from three independent experiments. *p < 0.05 versus the PBS control group.

We next evaluated the cytotoxic effects of bortezomib and rosuvastatin on human myeloma cell lines (IM9 and XG‐1). Dose‐response curves generated over 24–72 h confirmed that both drugs reduced cell viability in a dose‐ and time‐dependent manner (Figure 2A–D). We then hypothesized that combining these agents might yield superior anti‐tumor activity. Treatment with subtoxic concentrations of bortezomib and rosuvastatin together resulted in significantly greater suppression of cell proliferation at 72 and 96 h compared to either monotherapy in both cell lines (Figure 2E,F).

Since CCL3 is a key cytokine in MBD, we investigated whether the combination therapy modulates its expression. Western blot analysis showed that a 48‐h treatment with either drug alone reduced CCL3 protein levels, but the suppression was markedly stronger with the combination (Figure 2G,H). Importantly, this in vitro finding was consistent with clinical data: MBD patients receiving bortezomib plus statins had significantly lower bone marrow CCL3 levels than those on bortezomib alone (Figure 1C), supporting the translational relevance of our cellular results.

### 3.3 Bortezomib and Rosuvastatin Co‐Treatment Suppresses Tumor Progression and Preserves Bone Integrity in a Myeloma Xenograft Model

**FIGURE 3:**
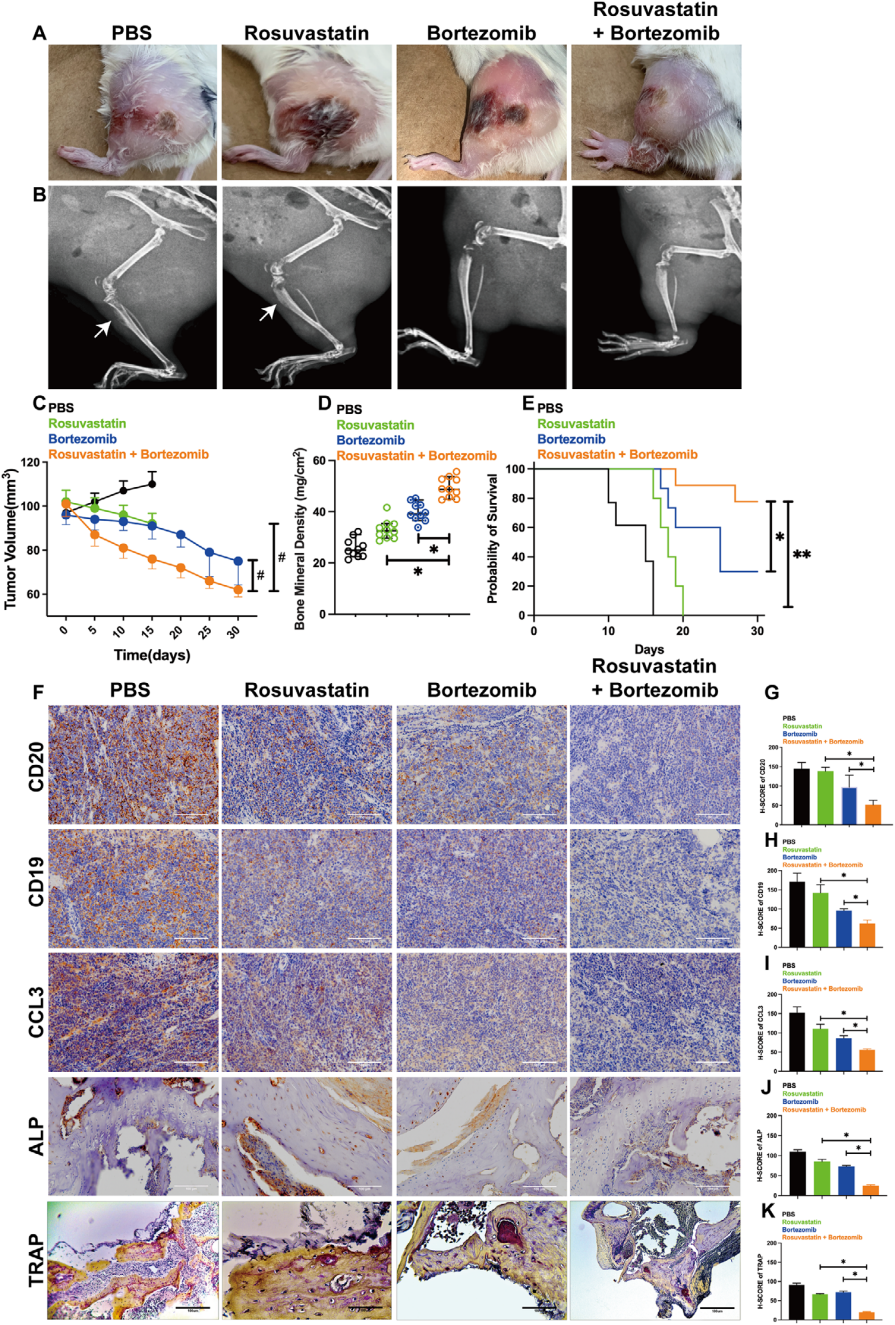
Bortezomib and rosuvastatin co‐treatment inhibits tumor growth and CCL3 expression in an IM9 xenograft mouse model. (A) Representative photographs of mice and corresponding x‐ray images of the hind limbs from each treatment group. Arrows in the x‐ray images indicate osteolytic lesions. The combination treatment group shows the smallest tumor size and the least bone destruction. (B) Tumor volume was measured over time in mice treated with PBS, rosuvastatin alone (20 mg/kg/day), bortezomib alone (0.5 mg/kg, twice weekly), or the combination of both drugs (*n* = 10 per group). Data are presented as mean ± SD. *p < 0.05. (C) Bone mineral density (BMD) of the femoral bones was measured by micro‐CT analysis at the endpoint. The combined treatment of bortezomib and rosuvastatin significantly inhibited the bone damage of MM. Data are presented as mean ± SD. *p < 0.05, combination group versus alone group. (D) Survival curves of NOD/SCID mice in the different treatment groups (*n* = 10 per group). The combination treatment significantly prolonged survival compared to all other groups (*p* < 0.05 by log‐rank test). (E) Representative immunohistochemical staining images of CD19, CD20, CCL3, ALP, and TRAP expression in bone marrow tissues from each treatment group. Scale bar, 100 µm. (G–K) Quantitative analysis of positive staining areas for (G) CD20, (H) CD19, (I) CCL3, (J) ALP, and (K) TRAP. Data are presented as mean ± SD. *p < 0.05, ** *p* < 0.01, # *p* < 0.001.

To validate these findings in vivo, we established an IM9 xenograft model in NOD/SCID mice. Representative photographs and x‐ray images showed severe osteolytic lesions in the PBS control group, while the combination treatment group exhibited the smallest tumor size and least bone destruction (Figure 3A).

Longitudinal monitoring revealed that the combination of bortezomib and rosuvastatin resulted in significantly stronger inhibition of tumor growth (Figure 3B), better preservation of femoral bone mineral density (Figure 3C), and a marked survival benefit (Figure 3D) compared to monotherapy or control groups.

Immunohistochemical analysis confirmed that the combination treatment most effectively reduced tumor burden (CD19/CD20 staining) (Figure 3E,G,H) and suppressed CCL3 expression in the bone marrow (Figure 3E,I). These in vivo findings demonstrate that the co‐administration of bortezomib and rosuvastatin synergistically inhibits tumor progression and preserves bone integrity.

### 3.4 The Combination Therapy Reverses CCL3‐Associated Bone Remodeling Imbalance In Vivo

Given the critical role of CCL3 in bone homeostasis, we further assessed its impact on OBs and OCs in vivo. Immunohistochemical analysis of bone marrow tissues showed that the suppression of CCL3 by the combination therapy was accompanied by a significant increase in ALP‐positive OBs (Figure 3E,J) and a marked decrease in TRAP‐positive OCs (Figure 3E,K).

These results align with our clinical observations that CCL3 levels negatively correlate with OBic marker bALP and positively with bone resorption‐related factor aCa (Figure 1D,E). Together, these data demonstrate that bortezomib and rosuvastatin co‐treatment not only inhibits tumor growth but also restores bone remodeling balance by downregulating CCL3.

## 4 Discussion

MBD is a common complication of MM^14^. It results from an imbalance in bone remodeling, characterized by increased osteolytic bone destruction without compensatory new bone formation [15]. Numerous studies have shown that MM cells can produce cytokines and growth factors, including IL‐1, IL‐3, IL‐6, TNF‐α, CCL3, and MIP‐1β, which affect OC formation and activity [16, 17, 18, 19]. CCL3, a member of the C‐C chemokine family [20], is recognized as a potent stimulatory factor for OCs in MM and is strongly associated with MBD [21].

In the present study, we evaluated the role of serum CCL3 in patients with MBD and found that CCL3 expression was significantly higher in MBD patients compared to control groups. Furthermore, our results indicated that CCL3 levels in the bone marrow increase with the progression of bone destruction. This positive correlation between CCL3 levels and the severity of osteolytic lesions aligns with and extends previous clinical observations [22, 23]. To elucidate the relationship between CCL3 and bone destruction, we analyzed the correlation of CCL3 with the biochemical markers bALP and aCa. Our findings demonstrated a positive correlation between CCL3 and aCa, as well as a negative correlation between CCL3 and bALP. In conclusion, we propose that CCL3 is secreted by MM cells, and that bone destruction mediated by CCL3 is associated with enhanced OC function and reduced OB activity.

Bortezomib, a proteasome inhibitor, has become one of the most important novel agents in the current therapy for MM. It has also been demonstrated that bortezomib is involved in the regulation of bone cell functions [24]. Metzler et al. demonstrated that bortezomib can inhibit osteoclastogenesis by inhibiting NF‐κB activation [25]. In addition, Zangari et al. observed an increase in ALP levels in a patient who responded positively to bortezomib treatment [26]. In another prospective study, Lund et al. treated newly diagnosed patients with four cycles of bortezomib and found that both bALP and procollagen type I N‐terminal propeptide (PINP) levels increased in patients who responded to the treatment [27]. These findings indicate that bortezomib directly stimulates OB growth and differentiation while inhibiting OC development and activity. Recently, researchers revealed that CCL3 levels were higher in patients who achieved complete remission (CR) compared to those with a response below CR. However, it has not yet been reported whether bortezomib affects the expression of OBs and OCs by inhibiting CCL3 expression. This study demonstrated that bortezomib exerts an inhibitory effect on MM cells and affects the secretion of CCL3 to varying degrees. Furthermore, we confirmed that changes in CCL3 expression can influence the levels of OBs and OCs. Overall, we propose that bortezomib may inhibit the progression of MBD.

Statins, which are inhibitors of the hydroxymethylglutaryl‐CoA (HMG‐CoA) reductase enzyme, are powerful cholesterol‐lowering medications that have significantly contributed to the prevention of cardiovascular disease [28]. Many studies have demonstrated that statins are effective not only in the treatment of hypercholesterolemia but also in the prevention of MM [29, 30, 31, 32, 33, 34]. In 2014, research revealed that statins can inhibit the mRNA and secretion of CCL3, as well as suppress the phosphorylation of extracellular signal‐regulated kinase 1/2 (ERK1/2) and Akt by inhibiting Ras prenylation in MM [9]. To evaluate whether statins affect OB expression and reduce OC levels, we treated MM cells with varying concentrations of rosuvastatin. The results indicated that rosuvastatin can inhibit the proliferation of MM cells and decrease CCL3 levels in vitro.

Particularly, the combination of rosuvastatin and bortezomib demonstrated a more significant inhibitory effect on MM cells. To further validate the in vitro findings, we constructed a mouse xenograft model. However, we found no significant difference in the therapeutic effect of rosuvastatin compared to the control group. Finally, we employed IHC to evaluate the levels of CCL3, OBs, and OCs. The results indicated that both bortezomib and rosuvastatin not only inhibited the secretion of CCL3 but also increased OB levels and decreased OC levels. The central role of CCL3 in coordinating such an imbalance is further supported by mechanistic studies linking it to the dysregulation of bone remodeling pathways [35, 36].

This rebalancing effect following CCL3 inhibition prompts consideration of its potential downstream effects on key signaling pathways. While the RANKL/OPG axis and Wnt signaling inhibitors such as Dkk‐1 and sclerostin were not directly measured here, investigating their interplay with CCL3 represents a crucial direction for future mechanistic studies.

## 5 Conclusions

Overall, we validated through both in vitro and in vivo experiments that bortezomib and rosuvastatin influence OB function and OC activity by inhibiting CCL3, thereby establishing a solid foundation for the treatment of MBD. However, the precise mechanisms by which CCL3 affects OB and OC activity remain unclear. In the future, we aim to elucidate how CCL3 influences the expression of OBs and OCs.

## Author Contributions

**Fujun Qu**: data curation, formal analysis, writing – original draft. **Mingxu Hui**: data curation, formal analysis, writing – original draft. **Fan Yang**: data curation, formal analysis, writing – original draft. **Bo Huang**: data curation, formal analysis. **Nian Liu**: data curation, formal analysis. **Yuchan He**: conceptualization and funding acquisition. **Xiaotao Wang**: funding acquisition, project administration and writing – review and editing.

## Funding

This study was supported by the National Natural Science Foundation of China (82560045) and Natural Science Foundation of Guangxi Zhuang Autonomous Region (2025GXNSFHA069032).

## Ethics Statement

The study was approved by the Ethics Committee of The Second Affiliated Hospital of Guilin Medical University.

## Conflicts of Interest

The authors declare no conflicts of interest.

## Data Availability

The data that support the findings are available from the corresponding author upon reasonable request.
